# Myelination- and immune-mediated MR-based brain network correlates

**DOI:** 10.1186/s12974-020-01827-z

**Published:** 2020-06-12

**Authors:** Manuela Cerina, Muthuraman Muthuraman, Marco Gallus, Nabin Koirala, Andre Dik, Lydia Wachsmuth, Petra Hundehege, Patrick Schiffler, Jan-Gerd Tenberge, Vinzenz Fleischer, Gabriel Gonzalez-Escamilla, Venu Narayanan, Julia Krämer, Cornelius Faber, Thomas Budde, Sergiu Groppa, Sven G. Meuth

**Affiliations:** 1 Department of Neurology with Institute of Translational Neurology, Münster University Hospital, Münster, Germany; 2 Movement Disorders, Imaging and Neurostimulation, Biomedical Statistics and Multimodal Signal Processing Unit, Department of Neurology, University Medical Center of the Johannes Gutenberg University, Mainz, Germany; 3 Departement of Radiology, University of Münster, Münster, Langenbeckstrasse 1, 55131 Mainz, Germany; 4 Institute of Physiology I, University of Münster, Münster, Germany

**Keywords:** Demyelination, Remyelination, Modularity, Network Dynamics, MRI

## Abstract

**Background:**

Multiple sclerosis (MS) is an autoimmune disease of the central nervous system (CNS), characterized by inflammatory and neurodegenerative processes. Despite demyelination being a hallmark of the disease, how it relates to neurodegeneration has still not been completely unraveled, and research is still ongoing into how these processes can be tracked non-invasively. Magnetic resonance imaging (MRI) derived brain network characteristics, which closely mirror disease processes and relate to functional impairment, recently became important variables for characterizing immune-mediated neurodegeneration; however, their histopathological basis remains unclear.

**Methods:**

In order to determine the MRI-derived correlates of myelin dynamics and to test if brain network characteristics derived from diffusion tensor imaging reflect microstructural tissue reorganization, we took advantage of the cuprizone model of general demyelination in mice and performed longitudinal histological and imaging analyses with behavioral tests. By introducing cuprizone into the diet, we induced targeted and consistent demyelination of oligodendrocytes, over a period of 5 weeks. Subsequent myelin synthesis was enabled by reintroduction of normal food.

**Results:**

Using specific immune-histological markers, we demonstrated that 2 weeks of cuprizone diet induced a 52% reduction of myelin content in the corpus callosum (CC) and a 35% reduction in the neocortex. An extended cuprizone diet increased myelin loss in the CC, while remyelination commenced in the neocortex. These histologically determined dynamics were reflected by MRI measurements from diffusion tensor imaging. Demyelination was associated with decreased fractional anisotropy (FA) values and increased modularity and clustering at the network level. MRI-derived modularization of the brain network and FA reduction in key anatomical regions, including the hippocampus, thalamus, and analyzed cortical areas, were closely related to impaired memory function and anxiety-like behavior.

**Conclusion:**

Network-specific remyelination, shown by histology and MRI metrics, determined amelioration of functional performance and neuropsychiatric symptoms. Taken together, we illustrate the histological basis for the MRI-driven network responses to demyelination, where increased modularity leads to evolving damage and abnormal behavior in MS. Quantitative information about in vivo myelination processes is mirrored by diffusion-based imaging of microstructural integrity and network characteristics.

## Introduction

Myelinated fibers in the white matter (WM) assure the effective communication between anatomical regions and essential influence brain function. Alteration in myelin composition in the WM pathways or in gray matter (GM) regions can lead to severe impairment of brain functioning [50]. WM and GM integrity relates to physiological functioning and mirrors pathological processes [30, 31, 38]. Addressing structural integrity with non-invasive magnetic resonance imaging (MRI) provides quantitative and correlative measures of tissue integrity. It robustly detects neuroinflammation and neurodegeneration as seen in multiple sclerosis [28, 32, 69]. However, it is not clear how microstructural integrity drives the entire network behavior and how it is related to histopathology and behavior. Diffusor tensor imaging (DTI) based on anisotropic water diffusion is a powerful method for non-invasive, highly sensitive estimation of WM structures in the brain, reflecting macroscopic axonal and myelin organization of fiber bundles [50, 63, 77, 85, 92]. Recent animal studies have shown that the myelin content of WM accounts to a larger extent for the variance of the DTI-derived fractional anisotropy (FA) scalar index [15], providing evidence that diffusion anisotropy measures in these regions are highly sensitive to myelination [15, 51, 86, 87]. Reconstructing network properties of the entire brain enable a robust characterization of alterations that are caused by pathological processes. In support of this view, analysis of network properties as derived from structural similarity measures in vivo showed network alterations (for WM and GM, [19, 64]) and associations between FA and level of demyelination, both for MS patients [36] and animal models of neuroinflammation [49]. In a similar manner, alterations of thalamocortical pathways were described for other diseases and suggest a more complex scenario than just the measurable morphological alterations [39, 46, 62, 68].

Pre-clinical studies have reported altered brain tissue properties in animal models of de- and remyelination [16, 41, 52, 90] and in models of experimental autoimmune encephalitis [25, 75]. Alterations followed specific regional patterns and occurred within specific temporal scales that were associated with periods of neuroinflammation [11, 13, 25, 75]. However, it remains unclear to which extent network characteristics mirror the level of de- and remyelination or inflammation. Moreover, it is not clear how network dynamics determine functional impairment and whether remyelination promotes complete recovery. We previously demonstrated that impaired cognitive function associated with demyelination was only restored by promoting remyelination when solely WM was affected, while de- and remyelination in cortical GM was still associated with functional alterations [12, 71]. Beside a clear spatial pattern, there seem to be mechanisms triggered by myelin loss that prevent full functional recovery.

To understand these dynamics—namely, when does myelin loss affect brain circuits at the network level and how it is related to behavioral performance—we make use of the cuprizone model of de- and remyelination. This is an animal model robustly used to study consequences of myelin loss under pathological conditions [17, 44]. Loss of myelin alters structure and architecture of neural networks, and hence may have a major impact on brain functioning [35]. The mechanisms underlying such alterations have been partially identified as altered distribution of ion channels following myelin loss [17, 44] and alteration of tissue excitability [11, 23, 24], with subsequent cognitive deficits [11, 12, 37, 80] but not obvious locomotor impairment [80].

Myelin loss persists as long as cuprizone is added to the diet. When cuprizone is omitted, spontaneous remyelination occurs and control-like levels of myelin are observed after only 3 weeks [11, 12, 65]. To closely track these dynamics on brain network characteristics and microstructural tissue properties, we investigated de- and remyelination processes in the cuprizone model at different time points by acquiring structural MRI data (including DTI), performing ex-vivo histopathology, and applying behavioral testing. Our data show that myelin loss and regain can be identified with immunohistological approaches, and the outcome matches results from in vivo structural MRI. While myelin levels follow the diet progression and cuprizone withdrawal afterwards, alterations of fractional anisotropy and network topology are associated with altered cognitive abilities which are still observed during remyelination and suggest permanent damage at the neuronal level [5, 7, 40].

## Material and methods

### Animals and experimental outline

The experiments were performed on C57BL6J mice (females, 9 weeks old at the beginning of treatment, Envigo, Rossdorf, Germany). Experiments were conducted in accordance with guidelines of local German authorities (LANUV ID: 84-02.04.2015.A585). All efforts were made to minimize stress for the animals in accordance with the ARRIVE guidelines [58]. Food and water were available ad libitum.

**Fig. 1:**
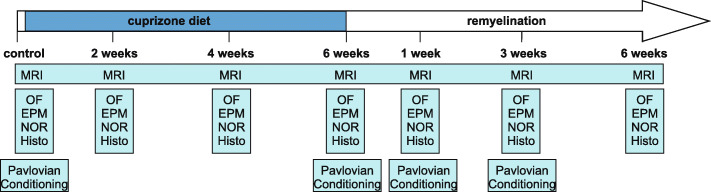
Experimental outline. Schematic representation of the study showing the 7 experimental groups coinciding with different time points before, during, and after cuprizone diet. MRI data was performed longitudinally at every time point in the same mouse cohort (continuous blue line). New cohorts of mice were used at each time points to assess locomotor-, anxiety-like behavior, and memory abilities. All mice underwent the same tests and some of them were used for histological evaluation at every time point. Additional cohorts were used to assess effects of Pavlovian conditioning paradigm only at the indicated time points

Cuprizone [bis(cyclohexylidenehydrazide)] was mixed with rodent pellet chow (0.2%). This compound is toxic for mature oligodendrocytes because it interferes with their internal mitochondrial metabolism and induces full demyelination after 6 weeks of diet. Omitting this compound from the diet allows spontaneous remyelination [65]. Mice were divided into 6 experimental groups: (i) 2, (ii) 4, and (iii) 6 weeks of cuprizone diet (model of demyelination, cupri 2, 4, and 6 weeks in the text), and 6 weeks of cuprizone diet followed by (iv) 1, (v) 3, and (vi) 6 weeks of normal food (model of remyelination, remy 1, 3, and 6 weeks in the text). A control group was matched for gender and age and received normal food for the whole duration of the study (see Fig. 1).

The same cohort of mice was longitudinally investigated by means of MRI and DTI. A new cohort of mice was used for each time point for behavioral tests (OF, EPM, and NOR) and some of the mice, as specified below, were used for ex vivo histological evaluation. A new cohort of mice was used to perform Pavlovian conditioning paradigms only in control conditions, after 6 weeks of cuprizone diet, at 1 week and 6 weeks of remyelination as shown in the experimental outline in Fig. 1.

### Immunohistochemistry

In order to evaluate the efficacy of the cuprizone diet, 3 to 10 animals which participated in behavioral tests were used for histopathological evaluation. Briefly, mice were deeply anesthetized using ketamine/xylazine and transcardially perfused using phosphate-buffered saline (PBS), as described before [12]. Afterwards, the brains were quickly removed, embedded in cryoprotective compound (TissuTeK, Science Service GmbH, Munich, Germany), and frozen using liquid nitrogen. Coronal cryosections (10-μm thickness) were cut using a cryotome (Leica), positioned on glass slides (two per slide) and conserved at −20 °C. Slices were fixed in a solution containing 4% paraformaldehyde (PFA) for 10 min and then washed with PBS. In order to avoid false-positive results, slices were incubated overnight at 4 °C with a blocking solution containing PBS, 0.03% Triton X-100, 10% goat serum, and 10% bovine serum albumin (BSA). After blocking, slices were incubated with the following primary antibodies: proteolipid protein (PLP, product number #9311, mouse anti-mouse, Abcam, Cambridge, UK, 1:250), a specific marker for myelin; glial fibrillary acidic protein (GFAP, product number #7260, rabbit anti-mouse, Abcam, 1:1000), a specific marker for astrocytes; and the amyloid precursor protein (APP2452S, rabbit anti-mouse, Cell Signaling, New England Biolab GmbH, Frankfurt, Germany, 1:100), a specific marker for neuronal and axonal damage. Antibodies were diluted in a cold solution containing 10% goat serum, 10% BSA, and PBS. Overnight incubation followed. Slides were then incubated for 1 h with the fluorophore-conjugated secondary antibody Cyanine Cy™2 for APP (goat anti-rabbit IgG, #111-225-144, *A* = 492 nm, *E* = 510 nm, Jackson ImmunoResearch Inc., West Grove, PA, USA, 1:300). Finally, the mounting medium Fluoromount-G™ containing DAPI (#00-4959, Invitrogen by Thermo Fisher Scientific, San Diego, CA, USA) was applied as marker for cell nuclei. To visualize PLP and GFAP, a 3,3-Diaminobenzidine (DAB)-based protocol was used according to the manufacturer’s instructions. Briefly, the secondary antibody (Biotin-conjugated goat anti-mouse IgG, DAB-87582, dianova GmbH, Hamburg, Germany) was applied at room temperature and incubated for 1 h. Then the avidin-biotin complex interaction method was used to specify the signal. For this purpose, the Vectastain A + B set (PK-6100, Vector Laboratories, Burlingame, USA, 1:100) and tris-buffered saline (TBS) were applied for 35 min [26].

### Immunohistochemistry analysis

Images were acquired using a Zeiss Examiner microscope. Images of slices containing the corpus callosum (CC), the neocortex (Cx), and the thalamus (Th) were collected from both hemispheres. A maximum of 11 slices per mouse were analyzed and considered as technical replicates for analysis of CC and Cx while more slices were used for analysis of the thalamus. All image analyses were performed in a blinded manner using ImageJ [78]. For PLP staining, images collected at a magnification of 5- and 10-fold were used for analysis. Myelin intensity, corresponding to intensity of DAB staining, was used as read-out and compared to control for all other experimental groups. For GFAP staining, images were acquired using 20- and 40-fold objectives and analyzed by counting the number of DAB positive cells per mm^2^. For APP, images were acquired using a 20-fold objective and analyzed by counting the number of fluorescence positive cells per mm^2^.

### Behavioral tests

Mice underwent a series of tests to evaluate locomotor activity, anxiety-levels, and cognitive performance.

#### Locomotor activity

The open field (OF) test was applied in order to evaluate locomotor activity and basal exploratory behavior. Animals (*n* = 10) were tested in the OF arena (35 × 40 × 40 cm). The distance covered and time spent in the periphery were taken as a read-out (Noldus Ethovision, The Netherlands).

#### Characterization of anxiety-like behavior

Animals (*n* = 10 for the control group and *n* = 5 for the other groups) were tested in the Elevated Plus Maze (EPM, Ethovision, Noldus IT bv, Wageningen, The Netherlands) to assess anxiety-like behavior. The EPM system is elevated from the floor (50 cm) with two closed and two open arms which the animal is allowed to explore for 5 min. Each group was tested once, and time spent in closed and open arms was taken as read-out.

#### Auditory Pavlovian conditioning

A modified auditory fear-conditioning paradigm was used, as described previously [12, 18, 72]. Mice (*n* = 5) were familiarized with the fear-conditioning apparatus (TSE System GmbH, Bad Homburg, Germany) twice during day 1 (with a 6-h interval) while being exposed to six neutral tones (unconditioned stimulus CS^−^, 2.5-kHz tone, 85 dB, 10-s duration; referred to as non-relevant stimulus in the text). On the next day, animals were exposed to the conditioned stimulus (three trials; CS^+^, 10-kHz tone, 85 dB, 9-s duration) randomly coupled with a mild foot shock (0.4 mA, 1-s duration, onset with CS termination). Then, 24 h after the last tone presentation, animals were again randomly presented with two tones, and freezing was taken as behavioral read-out. Freezing is the duration of immobility of the animal (except for respiratory movements) in response to presentation of the conditioned stimulus (10 kHz), as described previously [12, 18, 72].

#### Short- and long-term memory skills

Novel Object Recognition (NOR) was performed to evaluate cognitive and memory skills of the animals (*n* = 10; all time points; Ethovision, Noldus IT bv, Wageningen, The Netherlands). We used the same arena as for the OF test, since the animals were already familiar with it. The NOR test consists of a habituation phase during which animals are allowed to explore two identical objects for 10 min, followed by three retrieval phases performed at different time intervals after habituation in order to evaluate short non-hippocampal-related (15 min), short hippocampal-related (4 h), and long-term memory (24 h) skills [45]. For each retrieval session, one of the old objects was substituted for a novel one (chess pieces were used for all tests). Time spent exploring novel and old objects was used to calculate a NOR index [54] as follows: (time novel)/(time novel + time old). An index > 0.5 indicates that animals spent more time exploring the novel object than the old one, suggesting proper memory skills; an index = 0.5 indicates that animals spent an equal amount of time exploring the novel and old object, suggesting their inability to distinguish between the novel and the old [2].

A new cohort of mice was used for each of the seven time points described above in order to avoid learning effects, and they underwent OF, EPM, and NOR testing. Additional new cohorts of mice were used for Pavlovian conditioning paradigm which was only performed in control mice, after 6 weeks of cuprizone diet, 1 week, and 3 weeks after reintroduction on normal food.

### MRI and DTI

MRI was performed using a 9.4-Tesla small animal MR scanner with a mouse brain surface coil (Bio-Spec 94/20; Bruker BioSpin MRI GmbH, Ettlingen, Germany). Mice (*n* for each group is given in the results) were anesthetized in a warmed plexiglas box with 5% Isoflurane (Baxter, Germany) in 1 L/min O_2_. Isoflurane dosage was reduced to 1–1.5% in 1 L/min O_2_/compressed air 30/70 vol% for positioning in the animal cradle and subsequent scanning. Stable physiology was controlled by continuous monitoring of body temperature via a rectal temperature probe (36.5 ± 0.5 °C) and respiration rate (80–100 breath/min). Total examination time did not exceed 70 min.

We obtained T2-weighted (T2w) rapid acquisition with relaxation enhancement anatomical images. Diffusion tensor data were acquired with an eight-segment echo planar imaging (EPI)/diffusion tensor imaging (DTI) protocol (repetition time/echo time, 5000/30 ms, slice thickness 0.3 mm (20 slices), matrix size (128 × 128) resulting in an in plane resolution of 125 μm^3^) with *b* = 0, 1000 s/mm^2^ (30 diffusion directions, five B_0_ images, diffusion gradient duration of 5 ms, and diffusion gradient separation of 11 ms).

In accordance with the FSL (www.fmrib.ox.ac.uk/fsl) DTI pipeline, and after pre-processing for artefact correction (eddy currents and head movements), individual masks were generated for each mouse brain using the Brain Extraction Toolkit (BET) to isolate the brain from the skull. These masks were subsequently edited manually to correct for errors remaining from the masking process. A study-specific high-resolution mouse template was generated by nonlinearly registering each mouse brain to a single reference image. Further, output of non-linear registration was validated by checking for correct alignment of the surface of the brain and internal alignment of anterior commissure, corpus callosum, and cerebellum. An averaged image of the registered brains was constructed and used as reference image for subsequent analyses, and transformation matrices were generated between the reference image and individual mouse brain data sets using FNIRT (FSL, www.fmrib.ox.ac.uk/fsl) [42]. Fractional anisotropy (FA) values were obtained using FSL (http://www.fmrib.ox.ac.uk/fsl/). The detailed protocol is explained elsewhere [84]. In addition, distribution of crossing fibers was estimated using BEDPOSTX (implemented in FSL), and probability of major (f1) and secondary (f2) fiber directions was calculated [4]. Tractography was then computed for each voxel within the seed mask (using *n* = 5000 streamline fibers/voxel and curvature threshold of 0.2) and back-transformed into high-resolution mouse-standard space [53]. Allen mouse brain atlas (AMBA) with 82 identified brain regions was used [49, 61]. Thus, obtained fiber tracts were mapped to the FA skeleton for each animal to obtain FA values in the tracts between ROI’s [74]. Hence, a final 82 × 82 connectivity matrix was estimated based on the correlation of mean FA values of fiber tracts between those regions for each subject.

The obtained connectivity matrices were then investigated using the graph theoretical network framework to obtain various local and modular measures which would describe the topological reorganization of structural networks associated to the histological markers and behavior changes. The networks were then obtained at 20 different network densities, and the measures were extracted at each density. In graph theory analyses, the density represents cost of the network computed by fraction of present connections to all possible connections [47]. Hence, the network measures derived at each density for each time point would specify the alterations in network behavior between these time points at different levels of network fragmentation (from full, partial to discontinuous connectivity). This method of thresholding ensures that all the regions (nodes) of the network are connected while discarding spurious connections (edges) [1]. The local network measure was observed by computing “clustering coefficient” [91], and the modular reorganization was observed using “modularity” [34, 73]. All these measures were computed using BCT toolbox [76]. It has been shown that addressing both of these variables gives a robust conceptual characterization of the network characteristics at different systemic ranges [29]. Statistical analysis of the imaging data was performed in a blind manner; only preprocessing steps were performed in a non-blind manner.

### Statistics

All data are presented as means ± standard error mean (SEM) or medians with ranges. Analyses of variance (ANOVA) and covariance (ANCOVA) were performed using one way or factorial models (ANOVAs) followed by Tukey’s or Bonferroni’s post hoc tests (SPSS, IBM). Statistical analysis of histological results was performed using nested ANOVAs performed with GraphPad (Prism 8) in order to take into consideration technical replicates and biological ones and test for random effects. The other statistical analyses were performed using IBM SPSS Statistics, Version 22.0 (SPSS, Chicago, IL, USA) and GraphPad (Prism 5). Graphs were produced using Prism 5, and figures were created with Coreldraw x8.

## Results

### Time-dependent white matter de- and remyelination in the cuprizone model

**Fig. 2:**
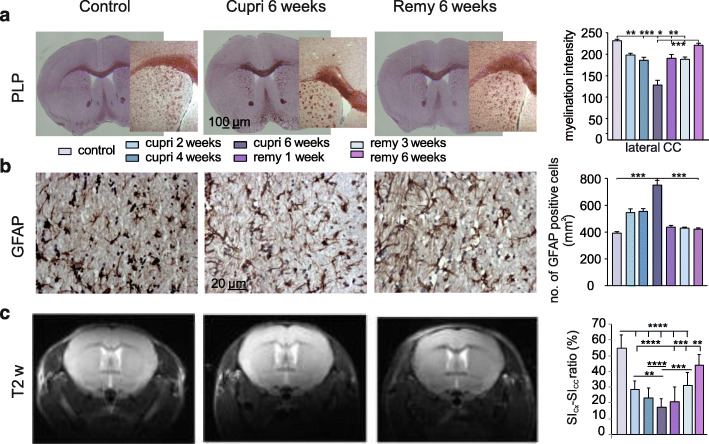
Structural and anatomical white matter changes during de- and remyelination in the cuprizone model. Example immunohistochemical images of coronal mouse slices containing the corpus callosum (CC) in control conditions (1st column), at 6 weeks after starting the cuprizone diet (cupri 6 weeks—full demyelination, 2nd column), and at full remyelination 6 weeks after reintroduction of normal food (3rd column). On the right of each panel, bar graphs show quantifications of changes. Stained for: **a** myelin specific marker PLP; **b** astrocytic specific marker GFAP; **c** exemplary T2-weighted images obtained in living mice during a longitudinal MRI scan. Pictures show frontal part of mouse brain containing neocortex and CC from control, cupri 6 weeks and remy 6 weeks mice. Bar graph shows the ratio calculated between the intensity of the myelin signal observed in Cx (SI_Cx_) and CC (SI_CC_). *p < 0.05; **p < 0.01; ***p < 0.001; ****p < 0.0001

Two weeks after the mice started on the cuprizone diet, the myelin intensity signal, which was evaluated in the corpus callosum (CC) using the specific marker PLP, was lower compared to control (198.2 ± 4.01 vs. 231.1 ± 4 respectively, nested ANOVA, F_(6, 26)_ = 19.55, *p* < 0.0001; Tukey’s post hoc test: cupri 2 weeks vs. control, *p* = 0.05; Fig. 2a and Supplementary Fig. 1a). The myelin intensity signal decreased constantly with continuation of the diet, reaching a minimum 6 weeks after starting the diet, evidencing large demyelinated white matter regions in the lateral part of the CC (127.9 ± 11.1, vs. control, *p* < 0.0001; Fig. 2a, middle column). In line with previous reports [65, 83], omitting cuprizone from the diet allowed remyelination: the myelin intensity constantly and significantly increased in comparison to the time point of maximal demyelination (cuprizone 6 weeks). Myelination values reached control-like levels 6 weeks after cuprizone withdrawal (221 ± 4.75; Fig. 1a, 1st and 3rd column and bar graph).

Demyelination was accompanied by activation of the innate immune system, namely by astrocytosis and microgliosis. In detail, the number of astrocytes observed in the CC was significantly higher in the presence of cuprizone already 2 weeks after starting the diet compared to control conditions (526.3 ± 21.6 cells/mm^2^ and 391.3 ± 9.6 cells/mm^2^, respectively; nested ANOVA, F_(6, 63)_ = 48.51, *p* < 0.0001; Tukey’s post hoc test, *p* < 0.0001; Fig. 2b). This number further increased 6 weeks after starting the diet (738.2 ± 24.55; *p* < 0.0001 vs. control, Fig. 2b). Interestingly, the number of astrocytes decreased during early and late phases of remyelination, almost reaching control-like values (remy 1 week 428.7 ± 13.9 cells/mm^2^; remy 3 weeks 421.2 ± 12.21 cells/mm^2^; remy 6 weeks 421.6 ± 7.78 cells/mm^2^; Fig. 2b).

Analyzing T2-weighted images evidenced a morphologically similar extent of demyelination in the CC, visible in the lateral part of this structure thereby validating our ex vivo experimental findings. Since myelin, given its lipid composition, appears hypointense in T2-weighted MR images, white matter regions appear darker than gray matter ones. Analysis of myelination intensity was carried out by calculating the ratio between the hyperintense signal from the cortex and the hypointense signal of the CC. Control conditions were characterized by a high ratio, namely, high differences between the two signals (54.9 ± 3.6, *n* = 5; Fig. 1d, first column and bar graph). Corroborating the histological results, the ratio calculated 2 weeks after starting the diet was already significantly lower than control (23.58 ± 1.8, one-way ANOVA, F_(6,57)_ = 29.31, *p* < 0.0001; Tukey’s post hoc test: *p* < 0.0001 vs. control, *n* = 12; Fig. 1d). The ratio did not significantly decrease any further 4 and 6 weeks after starting the diet (17.65 ± 1.8 and 20.87 ± 2.43, respectively, *p* < 0.0001 vs. control, *n* = 12; Fig. 1d). Allowing remyelination by re-introducing normal food into the diet induced an increase of the ratio at 1 week (*n* = 5) and 3 weeks of remyelination (*n* = 10), although values were still significantly different compared to control (*p* = 0.013 and *p* < 0.0001, respectively vs. control). Only at 6 weeks of remyelination values reached control-like levels (44.3 ± 2.03, *n* = 10; *p* = 0.085).

### Time-dependent gray matter de- and remyelination in the cuprizone model

**Fig. 3:**
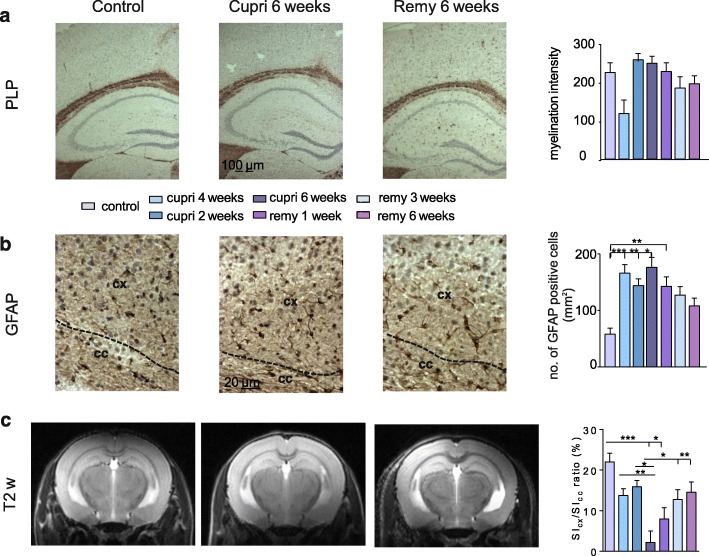
Structural and anatomical gray matter changes during de- and remyelination in the cuprizone model. Example immunohistochemical images of coronal mouse slices containing the neocortex (Cx) in control conditions (1st column), at 6 weeks after starting the cuprizone diet (cupri 6 weeks—full demyelination, 2nd column), and at full remyelination 6 weeks after reintroduction of normal food (3rd column). On the right of each panel, bar graphs show quantifications of changes. Stained for: **a** myelin specific marker PLP; **b** astrocytic specific marker GFAP; **c** exemplary T2-weighted images obtained in living mice during a longitudinal MRI scan. Pictures show frontal part of mouse brain containing neocortex, hippocampus, and CC. Bar graph shows the ratio calculated between the intensity of the myelin signal observed in caudal regions of the Cx (SI_Cx_) and CC (SI_CC_). *p < 0.05; **p < 0.01; ***p < 0.001; ****p < 0.0001

Next, in order to assess potential differences between white and gray matter regions upon cuprizone administration and withdrawal, we performed a structural analysis, similar to the one described above, but in the neocortex parenchyma (Cx). Already 2 weeks after starting the diet, myelin intensity evaluated in the Cx showed a tendency to be lower in comparison to control (117.5 ± 34.6 and 223.2 ± 24.41, respectively; nested ANOVA, F_(6,20)_ = 1.79, *p* = 0.15; Fig. 3a and e and Supplementary Fig. 2a). However, contrary to the CC, we did not observe large demyelinated areas, compared to control, at a similar time point (cupri 6 weeks 255.9 ± 20.07, Fig. 3a, middle column). This finding could be attributed to the low myelin content known to characterize some of the gray matter regions in comparison to white matter ones [59]. Similarly, no changes, compared to control, were observed during remyelination (remy 6 weeks 198.8 ± 22.3, left column; Fig. 3a). Astrocytosis and microgliosis were also observed in the neocortex (Fig. 3b). The number of astrocytes (control 57.94 ± 10.83 cells/mm^2^) was found to be significantly higher than control already 2 weeks after starting the diet (165.9 ± 15.1 cells/mm^2^; nested ANOVA, F_(6,20)_ = 4.86, *p* = 0.0036; Tukey’s post hoc test: control vs. cupri 2 weeks: *p* = 0.006; Fig. 3b and Supplementary Fig. 2b) and it remained relatively elevated at all investigated time points. The above same holds true for other gray matter regions like the somatosensory thalamus (Supplementary Fig. 3 and 4). Moreover, in order to evaluate potential neurodegeneration following oligodendrocyte and myelin damage, we also performed staining for amyloid precursor protein (APP), of which an accumulation in neuronal soma is considered an indicator for neurodegeneration [70]. Accumulation of APP was observed in the neocortex already at the beginning of the cuprizone diet and constantly increased at maximal demyelination but reaching significance threshold only during remyelinating phases (Supplementary Fig. 5). This would suggest that cuprizone and therefore demyelination is a strong insult to neurons.

Analyzing T2-weighted images in more caudal regions of the brains also evidenced a morphologically similar extent of demyelination in the CC, visible in the lateral part of this structure thereby validating our ex vivo experimental findings. Similarly to the condition observed for more rostral areas and corroborating the histological results, the ratio calculated 2 weeks after starting the diet was lower than control (13.74 ± 1.6, *n* = 12 and 22.04 ± 2.1, *n* = 5, respectively; Fig. 3d) and it reached significant threshold only 6 weeks after starting the diet (2.17 ± 2.8, *n* = 10; One-way ANOVA, F_(6,64)_ = 5.80, *p* < 0.0001; Tukey’s post hoc test: *p* < 0. 0001 vs. control; Fig. 3d). Interestingly, allowing remyelination by re-introducing normal food into the diet induced a significant increase in comparison to the full remyelination at 3 (12.76 ± 2.4, *n* = 10; *p* = 0.03) and 6 weeks of remyelination (14.53 ± 2.5, *n* = 12, *p* = 0.004). The MRI ratio analysis appeared to be more sensitive than histological analysis in this part of the brain.

### Behavioral correlates of general de- and remyelination

**Fig. 4:**
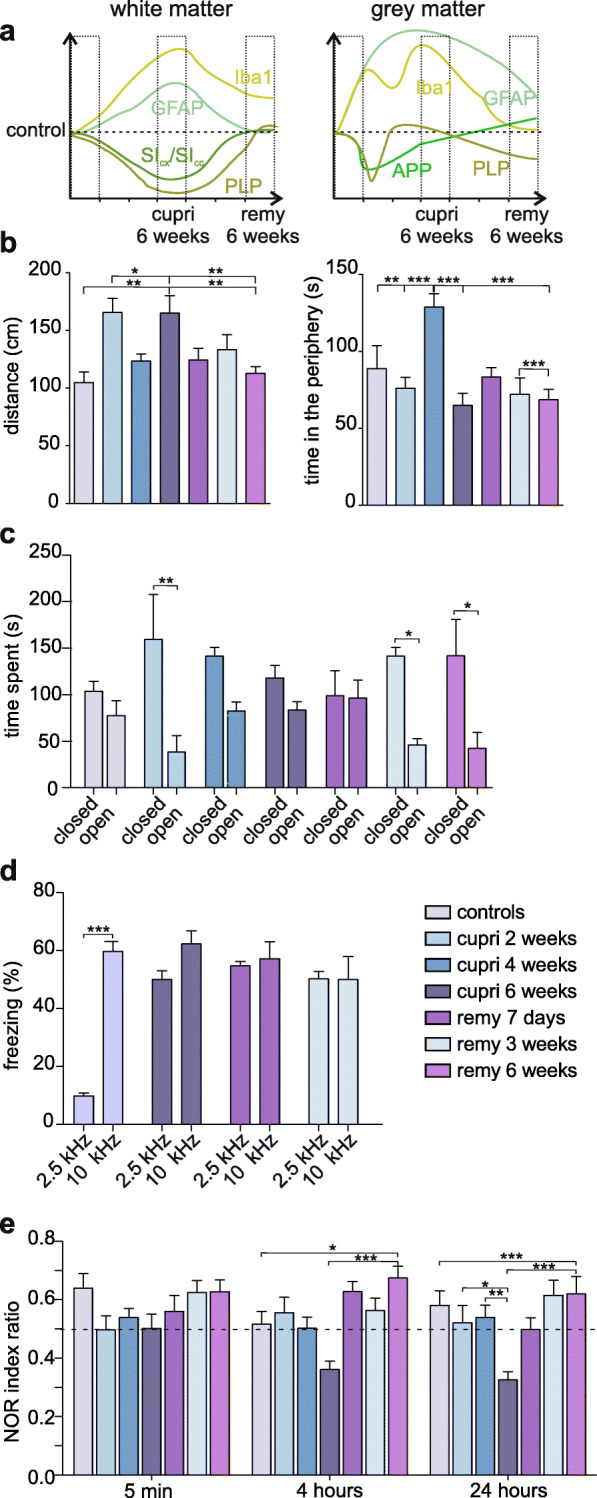
Behavioral correlates of general de- and remyelination in the cuprizone model. **a** Schematic representation of the time course of histopathological markers for structural impairment in the cuprizone model. **b** Bar graphs showing travelled distance and time spent in the periphery in the Open Field test. **c** Bar graphs show the results of the EPM test. **d** Bar graphs show the results of the auditory Pavlovian conditioning paradigm conducted using 2.5 kHz or 10 kHz, where the latter is the conditioning stimulus associated to the foot shock. **e** Bar graphs show NOR index calculated 15 min, 4 h, and 24 h. *p < 0.05; **p < 0.01; ***p < 0.001

Next, we performed behavioral experiments to investigate associations between the histological alterations and region-related functions such as cognition, anxiety-like behavior, and locomotor activity (Fig. 4). Therefore, in order to facilitate temporal presentation of the following behavioral data, a synopsis of histological evaluation in cortical white (Fig. 4a, left panel) and gray matter (Fig. 4a, right panel) is presented.

Animals tested in the OF 2- (165.6 ± 12.35 cm, Fig. 4b) and 6 weeks (165.1 ± 14.97 cm) after starting the cuprizone diet travelled a significantly longer distance in comparison to control (104.7 ± 9.24 cm, one-way ANOVA, F_(6,62)_ = 4.9, *p* = 0.0004; Tukey’s post hoc test: cupri 2 weeks and cupri 6 weeks vs. control, *p* < 0.01, Fig. 4b). This indicates locomotor hyperactivity of the mice, a behavior which was described previously [89] and seemed to be characteristic of demyelinated mice as animals from all remyelination time points travelled a similar distance as control animals (*p* = 0.016 vs. remy 6 weeks; Fig. 4b). To assess the level of anxiety-like behavior due to the new environment, we measured the amount of time animals spent in proximity of the walls and corners, both considered as shelter places. The group that received cuprizone for 4 weeks spent significantly more time in proximity to the walls (128.8 ± 8.64 s, Fig. 4b) compared with control animals (88.6 ± 15 s) and the other groups. This indicates anxiety-like behavior rather than locomotor impairment as there were no obvious differences in distances travelled (one way ANOVA, F_(6,62)_ = 5.82, *p* < 0.0001; Tukey’s post hoc test: cupri 4 weeks vs control: *p* = 0.048, vs. cupri 2 weeks: *p* = 0.002, vs. cupri 6 weeks: *p* < 0.0001, vs. remy 1 week: p = 0.012, and vs. remy 3 and 6 weeks: p < 0.001, Fig. 4b). Further analysis of number of vertical exploratory behaviors corroborated the hyperactivity and tendency to anxiety-like behavior as it was in general increased at any point of the diet and it is withdrawn in comparison to control (Supplementary Fig. 6a). In line, the grooming behavior, indicating the level of stress to which rodents might be exposed [55], it was significantly reduced in all experimental groups in comparison to control (Supplementary Fig. 6b).

To further interpret these findings, we performed the EPM test which is based on the normal exploration attitude of mice. Non-anxious, control mice spent an equal amount of time in the two arms (open 77.6 ± 15.9, closed 103.7 ± 10.6, Fig. 4c), while the animals on a 2-week cuprizone diet spent most time in closed arms (159.4 ± 48.3 s vs. open arms, 38.5 ± 17.7 s, two-way ANOVA, effect of the anxiety F_(1,66)_ = 31.7, *p* < 0.001; Tukey’s post hoc test: cupri 2 weeks open vs. closed arms: *p* < 0.01, Fig. 4c). Similar anxiety-like behavior was observed during the first 3 weeks of remyelination (closed arms 141.37 ± 9.7 s), and still persisted after 6 weeks of remyelination (closed arms 141.8 ± 3.9 s).

Next, we assessed the cognitive and learning abilities of the mice. Our previously published experiments [11, 12] demonstrate that demyelination heavily impairs the ability to retrieve information from different brain regions. Here, we chose the auditory Pavlovian conditioning paradigm, which, in accordance with our previous findings, revealed that cuprizone-treated mice fail in associating a specific tone frequency with aversive stimuli (Fig. 4d). Moreover, animals tested during early and late stages of remyelination (freezing at 2.5 kHz, 50 ± 2.9% and freezing at 10 kHz, 62.3 ± 4.5%; two-way ANOVA, effect of the diet, F_(1,32)_ = 28.3, *p* < 0.0001, Tukey’s post hoc test 2.5 kHz vs. 10 kHz, not significant, Fig. 4d) showed the same outcome as control mice (freezing at 2.5 kHz, 9.8 ± 1.03% and freezing at 10 kHz, 59.7 ± 3.4%; Tukey’s post hoc test 2.5 kHz vs. 10 kHz, *p* < 0.001; Fig. 4d), confirming that myelin loss triggers mechanisms that alter neuronal circuits associated with tone frequency discrimination as well as fear learning and memory. The impairment persisted after 3 weeks of remyelination (Fig. 4d). On this basis, we applied another well-established method to investigate short- and long-term memory abilities: the novel object recognition (NOR) test. Test was performed 15 min and 4 h after adaptation to test short-term memory and 24 h to assess long-term memory. Control animals recognized the novel object at all of the chosen time intervals, while impaired memory skills seemed to arise with diet onset and constantly progressed to an inverted performance 6 weeks after starting the diet (cupri 6 weeks: 24 h, 0.33 ± 0.03, two-way ANOVA, effect of the diet: F_(6,165)_ = 7.87, *p* < 0.0001; Fig. 4e). After reintroduction of normal food, the performance of mice ameliorated, reaching control-like values for short-term memory intervals already in the first week of remyelination, and for long-term memory intervals in the third week of remyelination. Taken together, our data suggest that improvement of memory abilities goes hand in hand with spontaneous remyelination.

### DTI analysis depicts altered FA values while the network analysis shows a regional discrepancy between the cortex and thalamus

**Fig. 5:**
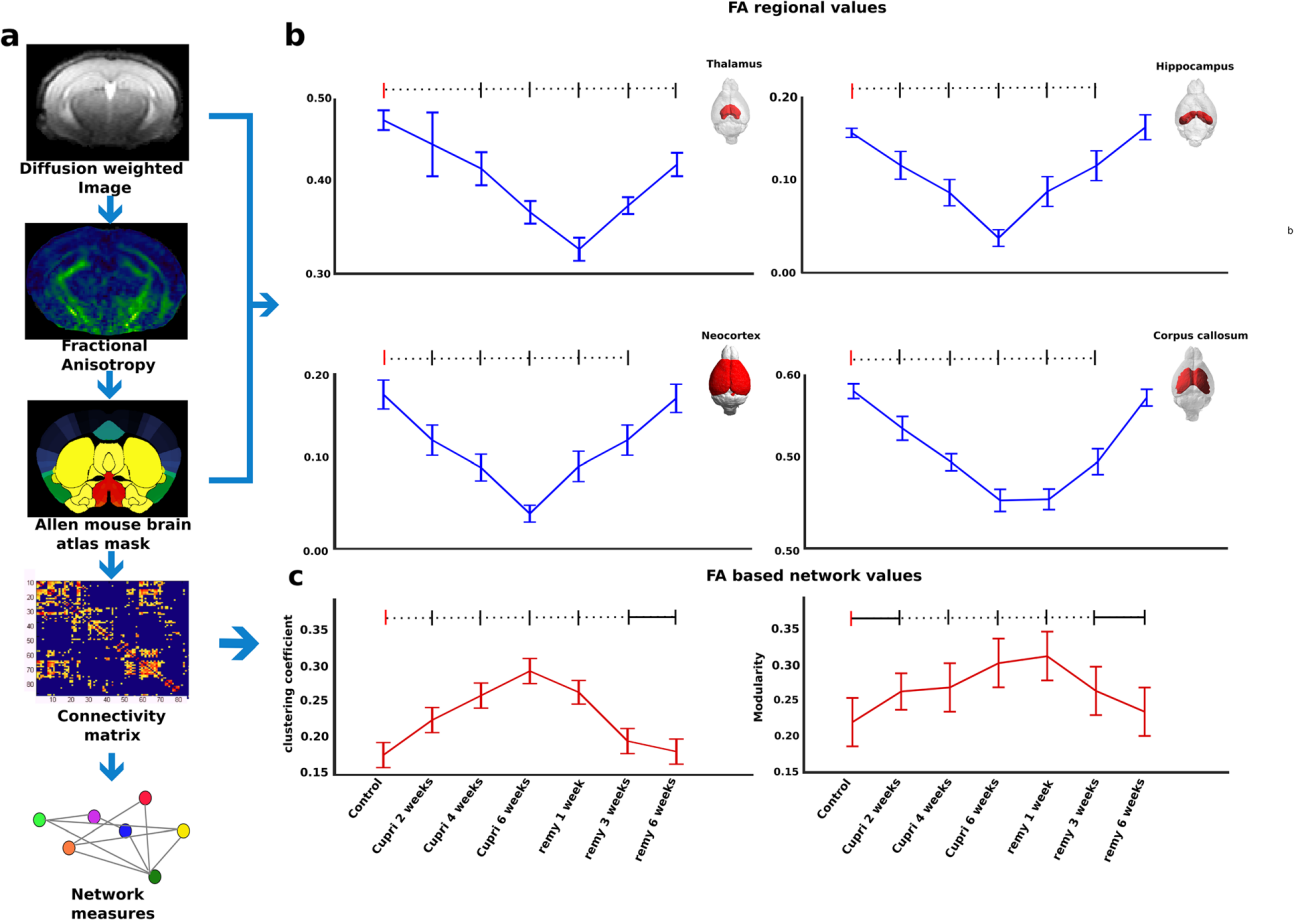
Structural dynamics of de- and remyelination. **a** Schematic representation of the methodological approach used to perform neuronal network analyses. Diffusion tensor images were used to calculate the fractional anisotropy (FA), and a connectivity matrix was built with the help of an anatomical mask. **b** Regional FA values in the thalamus (left upper panel), in the neocortex (left lower panel), and the hippocampus (right upper panel at all and CC (right lower panel) investigated at all-time points). **c** FA-based network values had increased clustering coefficients and modularity compared to control. Horizontal black lines represent significance of a given time point in comparison to control: *p* < 0.01. Dashed lines represent significance of a given time point in comparison to control: *p* < 0.001

The structural MRI data were further evaluated to analyze the FA and to build structural similarity maps for the entire brain. T2-weighted MR images and DTI-related parameters such as FA were extracted to measure structural similarity of the ROIs from the anatomical atlas. The cortex and hippocampus showed reduced FA values indicating a demyelination over time effect 6 weeks after starting the diet compared to control (one-way ANOVA, thalamus—F_(6, 83)_ = 5.81, *p* < 0.001; hippocampus—F_(6, 83)_ = 4.74, *p* < 0.001; cortex—F_(6, 83)_ = 6.23, *p* < 0.001; corpus callosum—F_(6, 83)_ = 4.98, *p* < 0.001; Fig. 5b). The post hoc comparisons between the other time intervals for all four regions were significant (*p* < 0.001) except for thalamus at cuprizone 2 weeks, and for the other three regions at the remyelination 6 weeks as shown in Fig. 5b with dashed line. In the cortex and hippocampus, FA recovered with reintroduction of normal food, following a similar time course as observed for myelin markers (Fig. 5b). However, analysis of thalamic FA indicated that this region only shows very low FA values during early remyelination, indicating a network abnormality in comparison to neocortical integrity. Addressing the network properties, we depict increased global clustering (Fig. 5c, left), and increased modularity over time (Fig. 5c, right) shows higher short-range connections, while compared to controls (for one-way ANOVA: clustering, F_(6, 139)_ = 27.2, *p* < 0.001; modularity, F_(6, 139)_ = 22.3, *p* < 0.001). The post hoc comparisons between the control and the other time intervals for both parameters were significant (*p* < 0.001) except for the clustering coefficient between remyelination 3 and 6 weeks (*p* < 0.01), and the modularity between control and cuprizone 2 weeks (*p* < 0.01) and between remyelination 3 and 6 weeks (*p* < 0.01).

**Fig. 6:**
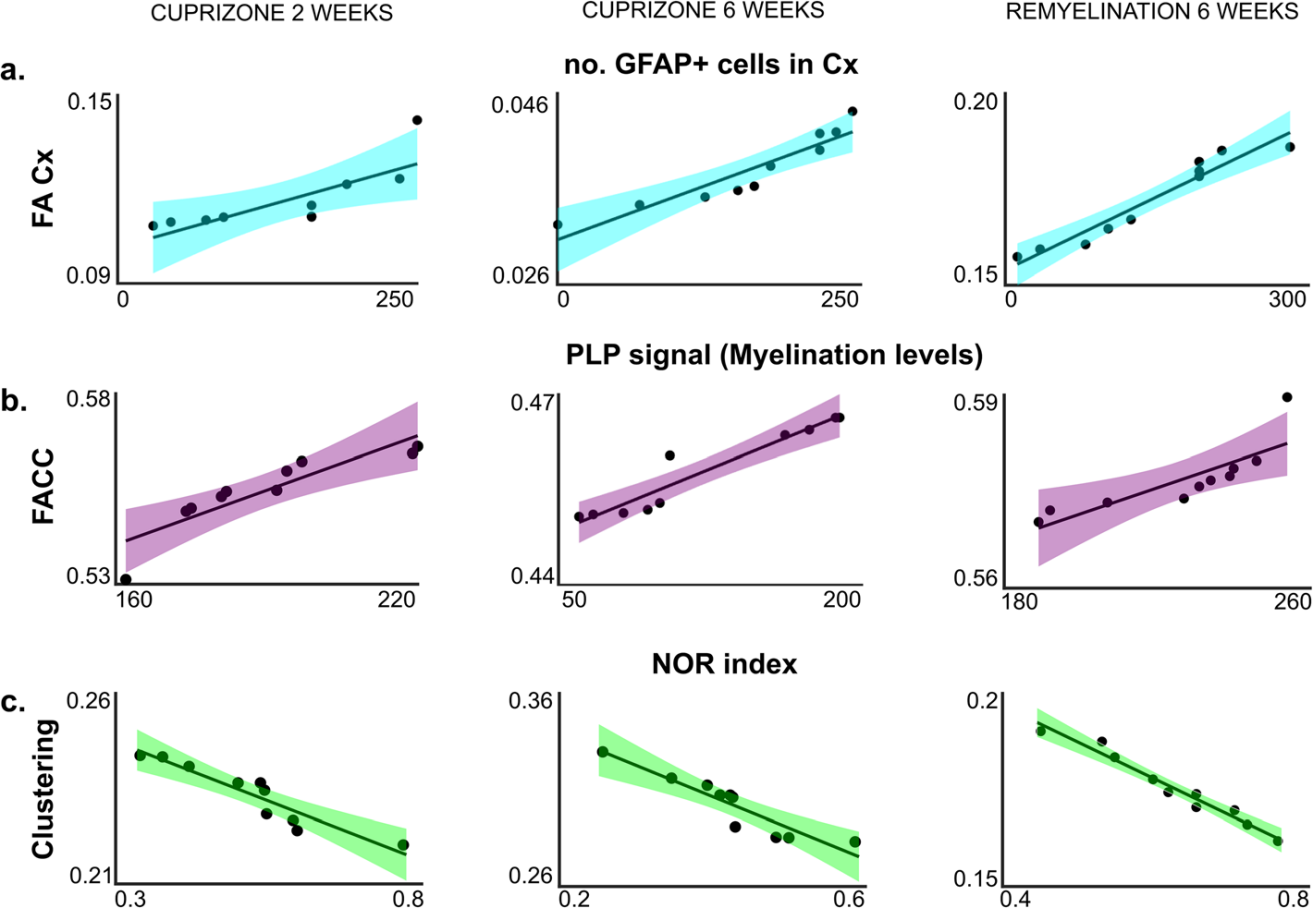
Correlation analyses. **a** Correlation plots between FA values in the cortex and number of GFAP positive cells found in the cortex. Three time points corresponding to onset of demyelination (cupri 2 weeks, left column), full demyelination (cupri 6 weeks, middle column), and full remyelination (remy 6 weeks, right column). **b** Correlation plots between FA values in the corpus callosum and PLP signal intensity (indication for myelination) in the corpus callosum. Three time points corresponding to the start of demyelination (cupri 2 weeks, left column), full demyelination (cupri 6 weeks, middle column), and full remyelination (remy 6 weeks, right column). **c** Correlation plots between clustering (indicator of network activity) and the NOR index. Three time points corresponding to the start of demyelination (cupri 2 weeks, left column), full demyelination (cupri 6 weeks, middle column), and full remyelination (remy 6 weeks, right column). *r*^2^ and *p* values are given in Table 1

So far, we investigated histopathological differences between white and gray matter regions in the cuprizone model of general de- and remyelination. Moreover, we linked these differences to specific behaviors and changes in brain connectivity observed with the help of in vivo approaches. As final step, we performed correlation analyses to further characterize the role of white and gray matter myelination in a disease model. A positive correlation was observed between the number of GFAP positive cells in the cortex and the FA values (Fig. 6a and Table 1), thus supporting the relation between astrocyte count and MRI-driven microstructural integrity or damage in the WM and GM regions [8]. We found a positive correlation between FA values measured in the CC and content of myelin for the studied groups at all-time points (Fig. 6b and Table 1).

**Table 1:** *r*^2^ and *p* values obtained from correlation analysis for all experimental groups and different parameters

**Correlation parameters (corrected r**^**2**^**)**		**Cupri 2 weeks**		**Cupri 4 weeks**		**Cupri 6 weeks**		**Remy 1 week**		**Remy 3 weeks**		**Remy 6 weeks**	
		***r***^**2**^	***p***	***r***^**2**^	***p***	***r***^**2**^	***p***	***r***^**2**^	***p***	***r***^**2**^	***p***	***r***^**2**^	***p***
**FA thalamus**	**Distance OF**	0.45	< 0.01	0.67	< 0.001	0.61	< 0.001	0.63	< 0.001	0.52	< 0.001	0.61	< 0.001
**FA hippocampus**	**Time in periphery**	0.58	< 0.001	0.62	< 0.001	0.64	< 0.001	0.56	< 0.001	0.58	< 0.001	0.64	< 0.001
**FA cortex**	**number of GFAP**^**+**^**cell in cortex**	0.62	< 0.001	0.63	< 0.001	0.57	< 0.001	0.53	< 0.001	0.62	< 0.001	0.58	< 0.001
**FA corpus callosum**	**PLP myelination**	0.59	< 0.001	0.64	< 0.001	0.62	< 0.001	0.59	< 0.001	0.57	< 0.001	0.64	< 0.001
**Clustering**	**NOR index**	0.48	< 0.01	0.59	< 0.001	0.62	< 0.001	0.56	< 0.001	0.59	< 0.001	0.65	< 0.001

Moreover, we investigated the correlation between clustering coefficients and behavioral variables. Clustering describes the networks at the local level and depends on their property to form microstructurally similar entities. Indeed, we observed a negative correlation between clustering and the NOR index 2 weeks after diet onset (Fig. 6c and Table 1).

## Discussion

Non-invasive investigation of the brain networks and studies on the relation of cerebral circuit characteristics and microstructural tissue properties are challenging since regional and global pathophysiological processes cannot be robustly delimited through widely available methodological tools. Here, we overcome this gap by linking histopathological analyses of myelin dynamics with diffusion MRI-derived metrics of tissue integrity and network characteristics (modularity, clustering) and dissect this essential pathophysiological bridge using the cuprizone model. Moreover, we relate these variables to function and psychopathology studying the behavioral correlates of the de- and remyelination.

Moreover, our study brings together several independent approaches that combined MRI and DTI technology during demyelination only [94] or focused solely on white matter regions with [93] studies investigating remyelination and effects in gray matter regions.

Our results indicate that histological and cytomorphological abnormalities in response to cuprizone-induced damage are closely interrelated to network properties as measured by diffusion MRI and are directly influenced (to a different extent) by processes occurring in both gray (cortex and thalamus) and white matter (corpus callosum). These structural reorganizations are caused by gradual myelin loss and occurrence of astrocytosis followed by remyelination processes. Our findings explicitly show how white and gray matter myelination differently affects microstructural integrity and network properties and how circuit variables parallelize improvement of behavioral performance and psychopathology. While control-like levels of myelin are observed during remyelination, cognitive function does not improve completely throughout remyelination. Hence, cuprizone-treated animals tested with a modified auditory Pavlovian conditioning paradigm were cognitively abnormal upon myelin loss and remained impaired in their performance even after reintroduction of normal food. Interestingly, in the same animals, we observed recovery of cognitive performance during remyelination in the memory task. This observation suggests network-specific effects: while hippocampal memory-related functions recover (this study), the auditory thalamocortical network, providing the anatomical basis for the sensory input for fear conditioning with its connection to the limbic system, remains affected (our previous studies and a study by [81]). Thus, we can hypothesize that persistent cognitive decline depends on alterations occurring at the network level and involves a modified balance of long- to short-range structural similarity dependent tissue reorganization (as shown by modularity alterations in our study). Moreover, the ability of neural circuits to re-wire and recruit higher numbers of neuronal populations or spatially separated neuronal populations, thereby interfering with a proper response to behavioral tasks, could be a potential mechanism underlying disturbed function [3, 9].

Reorganization of white matter pathways has been described as a potential mechanism to explain these effects [3, 57]. This explanation is supported by the results of our correlation analyses between FA values and PLP signal (Table 1) and by reduced excitability at maximal myelin loss probably due to lack of stimulus propagation [33, 44]. Interestingly, the cuprizone diet leads to a transitory period of hyperexcitability in early phases of remyelination associated with altered activity in the several network regions in vitro [11] and in vivo and affects white and gray matter regions differently [71]. Accordingly, we show that white and gray matter network properties as derived from fractional anisotropy measures clearly decrease during demyelination, with a subsequent increase upon remyelination.

Detrimental effects of myelin loss and inflammation have been shown for the neocortex in animal models of neurodegeneration [8, 49] and for sensory motor systems of MS patients ([20]; Fleischer et al., 2016). We could depict reduced FA values in the cortex and hippocampus, with a significant negative peak 6 weeks into the diet. Subsequently, FA values of the cortex and hippocampus increased with reintroduction of normal food, indicating neural tissue recovery following diet-induced damage and regain of baseline pre-cuprizone behavioral activity. Contrary, thalamus FA values remained very low during early remyelination, indicating that this anatomical structure is more susceptible to demyelination, showing slightly delayed effects compared to the cortex. This may directly influence the thalamocortical communications and the network metrics. These mechanisms could be responsible for the lost ability to differentiate auditory stimuli during demyelination and residual deficits with remyelination. Indeed, focal demyelination in the thalamus induces altered sensory responses to stimuli entering the auditory thalamocortical circuitry at a later time point of remyelination compared to the cortex [71]. Similarly, despite the amount of myelin produced by differentiated oligodendrocyte progenitors [83, 95], inter-hemispheric and interhemispheric connectivity through the CC is impaired then through cuprizone-induced demyelination and remains abnormal during remyelination. This supports the idea that myelin loss triggers a more permanent and profound decline of neuronal network functionality [11, 12, 17].

The increased clustering observed in MS patients compared to healthy controls represents a cost-efficient reorganization of the brain with amplified local information flow [29, 69, 82, 88]. In addition, increased modularity suggests a network reorganization with a modification of the long-range structural similarity and more local homogeneity in response to demyelination [29, 60, 69]. We observed an increase of modularity in the demyelination phase and an immediate reversal after stopping the cuprizone diet. These patterns of brain circuitry reorganization upon de- and remyelination follow a known scheme of brain circuits remodeling during brain development ([14]; Huang et al., 2013 [10];). Tissue microstructural abnormalities, indicated by FA dynamics in the course of the experiment, could be detected in gray matter regions only. Notably, network topology characteristics obtained from FA values could be detected at the global (whole brain) level, providing us with a more perceptible marker for ongoing structural changes. In contrast, our healthy controls showed lower clustering and lower modularity, demonstrating potentially mirroring aspects of compensation and adaptive reorganization in neighboring anatomical structures during early demyelination and remyelination phases. Several studies have presented evidence that links community structure properties of the brain (e.g., increased modularity) to maintenance of function despite continuous damage, as seen in neurodegenerative disorders [66]. These processes of network reorganization are presumably essential to maintain functioning [56, 67]. It is therefore important to consider that, apart from restored memory skills of mice in the NOR test, anxiety-like behavior in the EPM test and loss of frequency discrimination in the auditory Pavlovian conditioning paradigm were still present upon myelin gain. Similarly, the distance travelled in the OF test was also altered after myelin loss, supporting other recent findings [6]. The network-based approach applied here bridges ex vivo tracked tissue dynamics and in vivo microanatomy upon myelin loss and renewal, shaping discrete determinants of behavioral adaptive responses.

## Conclusion

Our data provide new links between histopathological myelin properties of the white and gray matter and brain circuit behavior at the network level as derived from MRI-driven diffusion imaging. We depict the basis for brain circuit modularization under demyelination and behavior abnormalities captured in a spatiotemporal manner. These translational concepts can be applied to address microstructural integrity, brain network responses, functional outcome to track disease courses in CNS autoimmunity, or therapeutic responses.

## Supplementary information

**Additional file 1: Figure S1.** Representation of technical and biological replicates for histological evaluation of PLP intensity, astrocytosis and microglial activation in the CC. (a) Scatter plots and graphs show the variability of the data acquired and used for histological evaluation on myelin intensity by using the specific marker PLP in the corpus callosum (upper panel). (b) Scatter plots and graphs show the variability of the data acquired and used for histological evaluation of the number of astrocytes by using the specific marker GFAP in the corpus callosum (mid panel). Simplified bar graphs are shown in the main figures.

**Additional file 2: Figure S2.** Representation of technical and biological replicates for histological evaluation of PLP intensity, astrocytosis and microglial activation in the Cx. (a) Scatter plots and graphs show the variability of the data acquired and used for histological evaluation on myelin intensity by using the specific marker PLP in the cortex (upper panel). (b) Scatter plots and graphs show the variability of the data acquired and used for histological evaluation of the number of astrocytes by using the specific marker GFAP in the cortex (mid panel). Simplified bar graphs are shown in the main figures.

**Additional file 3: Figure S3.** Structural and anatomical thalamic grey matter changes during de- and remyelination in the cuprizone model. (a) Exemplary pictures show staining for the specific myelin marker PLP in coronal mouse slices containing the ventrobasal complex of the thalamus (VB) in control conditions (left), at 6 weeks after starting the cuprizone diet (cupri 6 weeks – full demyelination, middle), and at full remyelination 6 weeks after reintroduction of normal food (right). Note the decreased signal for PLP indicating demyelination in the cupri 6 weeks group in comparison to control, and a persistent low PLP signal during remyelination. On the right, bar graphs show the quantification of myelin loss and regain for all groups and all investigated time points. (b) Exemplary pictures show staining for the specific astrocytic marker GFAP in coronal mouse slices containing the ventrobasal complex of the thalamus in control conditions (left), at 6 weeks after starting the cuprizone diet (cupri 6 weeks – full demyelination, middle), and at full remyelination 6 weeks after reintroduction of normal food (right). Note the increased number of astrocytes indicating astrocytosis in the cupri 6 weeks group in comparison to control and remy 6 weeks groups. On the right, bar graphs show the number of astrocytes (cells/mm2) in VB, this increased according to diet progression and cuprizone withdrawal.

**Additional file 4: Figure S4.** Representation of technical and biological replicates for histological evaluation of PLP intensity, astrocytosis and microglial activation in the thalamus. (a) Scatter plots and graphs show the variability of the data acquired and used for histological evaluation on myelin intensity by using the specific marker PLP in the thalamus (upper panel). (b) Scatter plots and graphs show the variability of the data acquired and used for histological evaluation of the number of astrocytes by using the specific marker GFAP in the thalamus (mid panel).

**Additional file 5: Figure S5.** The amyloid precursor protein (APP) accumulation upon de- and remyelination in frontal neocortical regions. Exemplary pictures show APP staining in the lower layers of frontal neocortical regions in control conditions (left), at 6 weeks after starting the cuprizone diet (cupri 6 weeks – full demyelination, middle), and at full remyelination 6 weeks after reintroduction of normal food (right). Note that an increase of positive cells, indicating an accumulation of APP in the neuronal soma, occurred slowly at the onset of the cuprizone diet to reach a significant threshold at remyelinating phases. On the right, bar graphs show the number of APP positive cells (cells/mm2) in Cx.

**Additional file 6: Figure S6.** Exploratory and grooming behavior were altered by cuprizone diet and its withdrawn. (a) Bar graph showing quantification of vertical exploratory behavior. Animals show a significant increase in comparison to control 2- and 6 weeks after the beginning of the diet. (b) Bar graph showing the quantification of grooming behavior. The latter is often considered a indicator of stress levels I rodents and here it is significantly decreased, in comparison to control, in almost all experimental groups. *p < 0.05; **p < 0.01; ***p < 0.001; ****p < 0.0001.

## Data Availability

The parts of the raw datasets are available in the link below due to upload space requirements. The complete dataset used and/or analyzed during the current study available from the corresponding author on reasonable request. The data used for creating the figures are available in the given link (https://www.dropbox.com/sh/xmxmzosxrudpx1l/AABM7L_jtkd2z6oi3pnqVNZoa?dl=0).
