# Supplementary figures and images for: Myelination- and immune-mediated MR-based brain network correlates
Source: J Neuroinflammation. 2020 Jun 12;17:186. doi: 10.1186/s12974-020-01827-z (PMC7293122; doi:10.1186/s12974-020-01827-z)

a

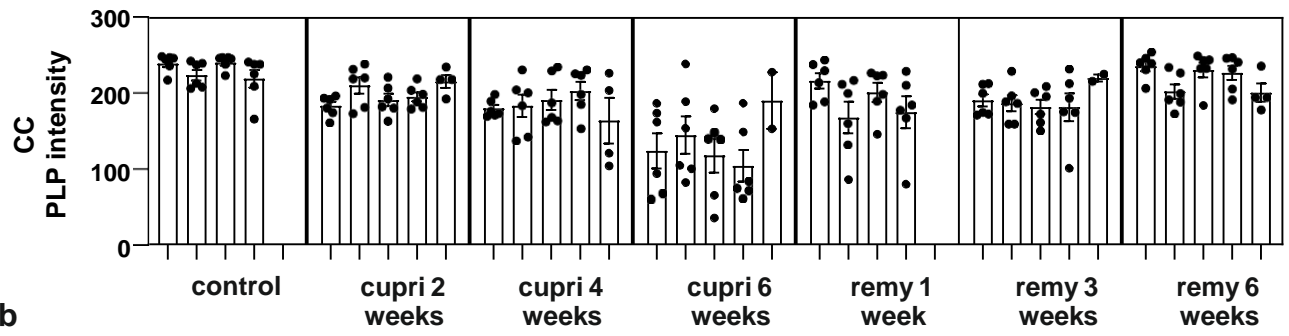

b

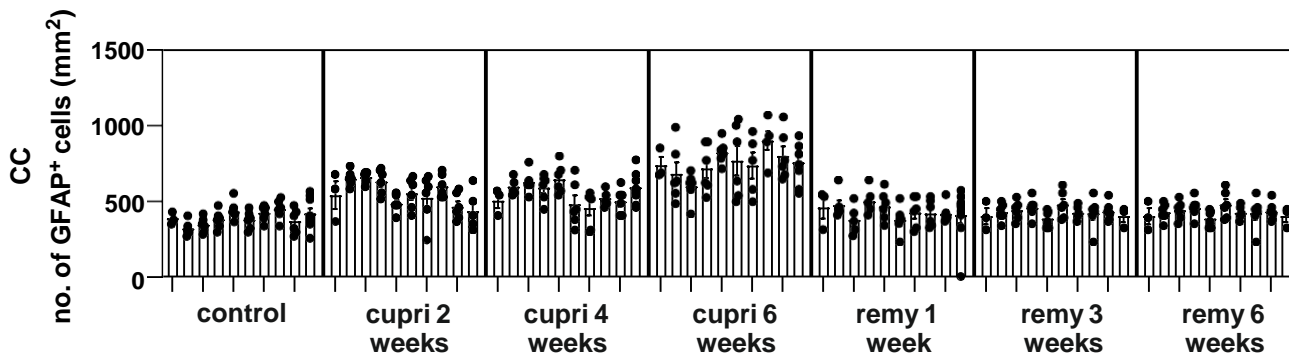

Supplement: Supplementary file 1 — Additional file 1: Figure S1. Representation of technical and biological replicates for histological evaluation of PLP intensity, astrocytosis and microglial activation in the CC. (a) Scatter plots and graphs show the variability of the data acquired and used for histological evaluation on myelin intensity by using the specific marker PLP in the corpus callosum (upper panel). (b) Scatter plots and graphs show the variability of the data acquired and used for histological evaluation of the number of astrocytes by using the specific marker GFAP in the corpus callosum (mid panel). Simplified bar graphs are shown in the main figures. [file 12974_2020_1827_MOESM1_ESM.pdf]

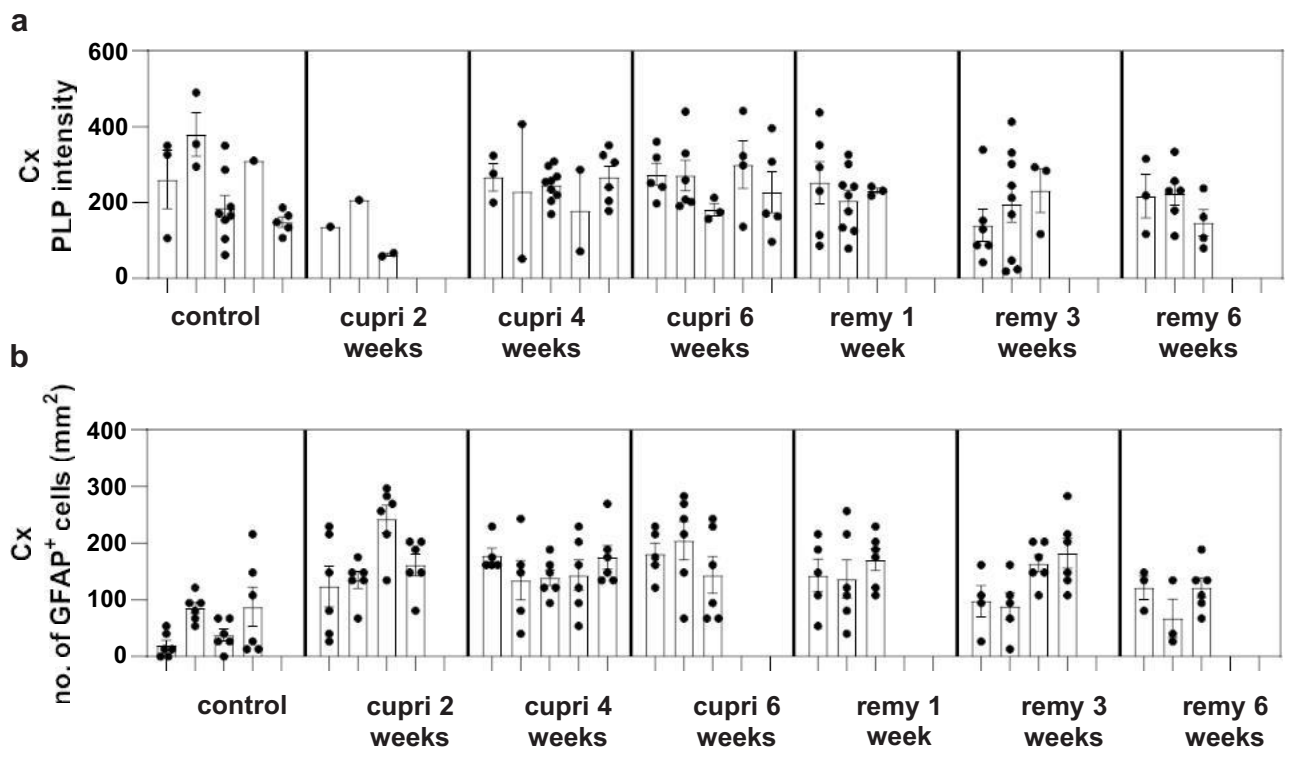

Supplement: Supplementary file 2 — Additional file 2: Figure S2. Representation of technical and biological replicates for histological evaluation of PLP intensity, astrocytosis and microglial activation in the Cx. (a) Scatter plots and graphs show the variability of the data acquired and used for histological evaluation on myelin intensity by using the specific marker PLP in the cortex (upper panel). (b) Scatter plots and graphs show the variability of the data acquired and used for histological evaluation of the number of astrocytes by using the specific marker GFAP in the cortex (mid panel). Simplified bar graphs are shown in the main figures. [file 12974_2020_1827_MOESM2_ESM.pdf]

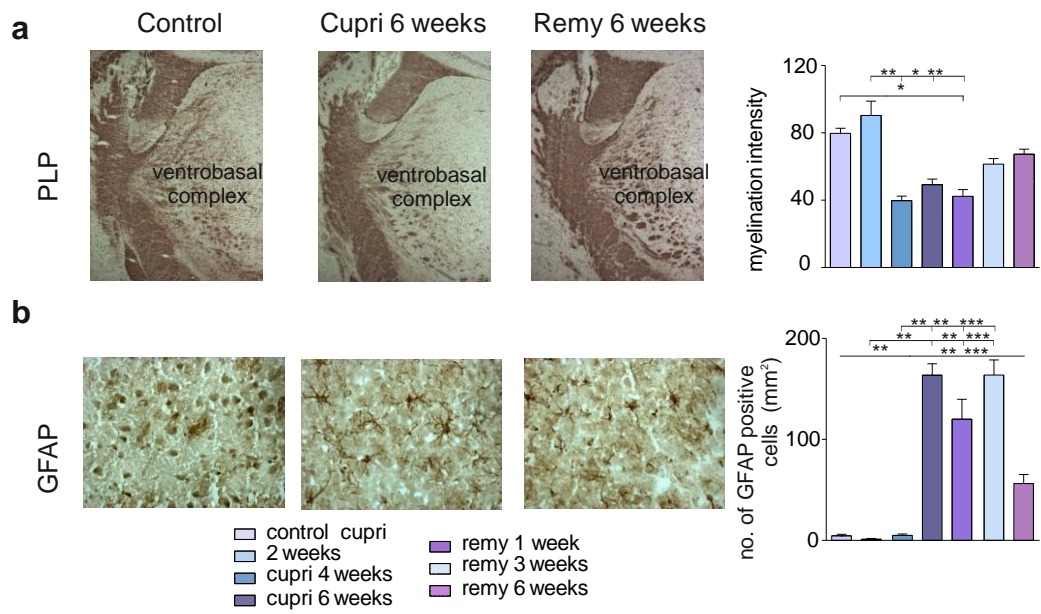

Supplement: Supplementary file 3 — Additional file 3: Figure S3. Structural and anatomical thalamic grey matter changes during de- and remyelination in the cuprizone model. (a) Exemplary pictures show staining for the specific myelin marker PLP in coronal mouse slices containing the ventrobasal complex of the thalamus (VB) in control conditions (left), at 6 weeks after starting the cuprizone diet (cupri 6 weeks – full demyelination, middle), and at full remyelination 6 weeks after reintroduction of normal food (right). Note the decreased signal for PLP indicating demyelination in the cupri 6 weeks group in comparison to control, and a persistent low PLP signal during remyelination. On the right, bar graphs show the quantification of myelin loss and regain for all groups and all investigated time points. (b) Exemplary pictures show staining for the specific astrocytic marker GFAP in coronal mouse slices containing the ventrobasal complex of the thalamus in control conditions (left), at 6 weeks after starting the cuprizone diet (cupri 6 weeks – full demyelination, middle), and at full remyelination 6 weeks after reintroduction of normal food (right). Note the increased number of astrocytes indicating astrocytosis in the cupri 6 weeks group in comparison to control and remy 6 weeks groups. On the right, bar graphs show the number of astrocytes (cells/mm2) in VB, this increased according to diet progression and cuprizone withdrawal. [file 12974_2020_1827_MOESM3_ESM.pdf]

**a**

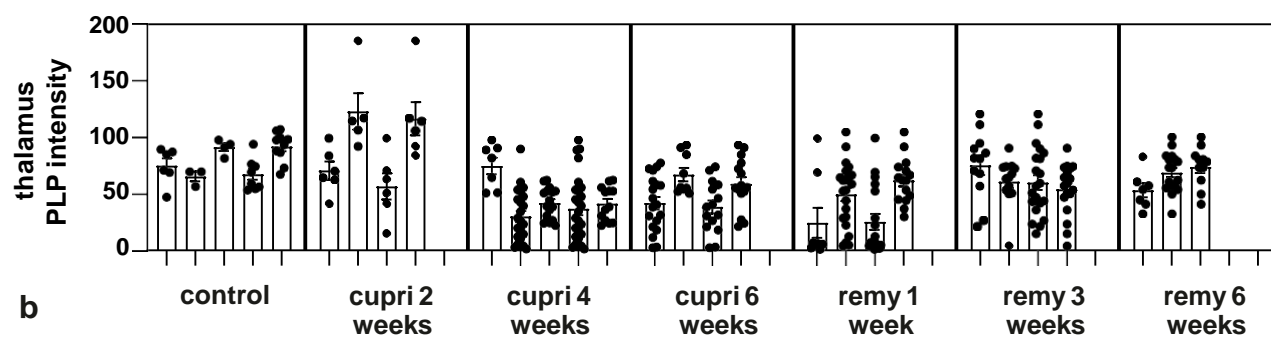

**b**

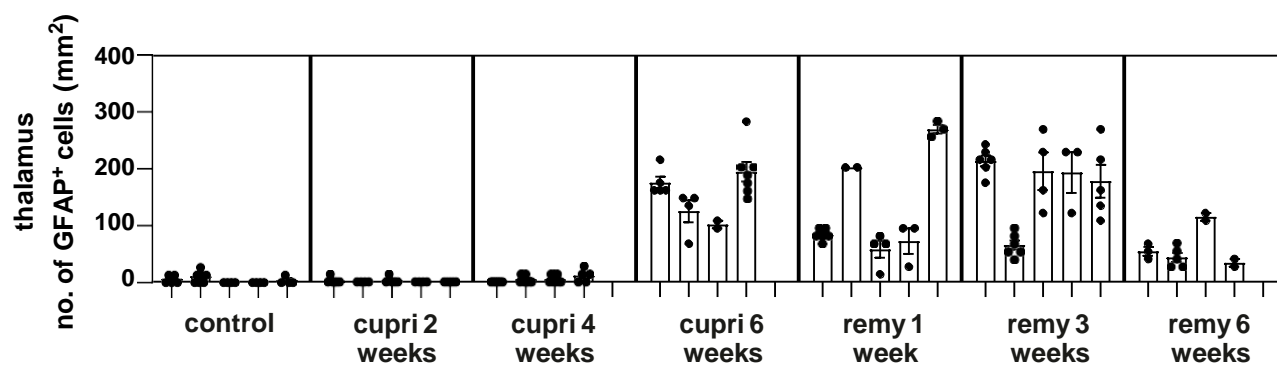

Supplement: Supplementary file 4 — Additional file 4: Figure S4. Representation of technical and biological replicates for histological evaluation of PLP intensity, astrocytosis and microglial activation in the thalamus. (a) Scatter plots and graphs show the variability of the data acquired and used for histological evaluation on myelin intensity by using the specific marker PLP in the thalamus (upper panel). (b) Scatter plots and graphs show the variability of the data acquired and used for histological evaluation of the number of astrocytes by using the specific marker GFAP in the thalamus (mid panel). [file 12974_2020_1827_MOESM4_ESM.pdf]

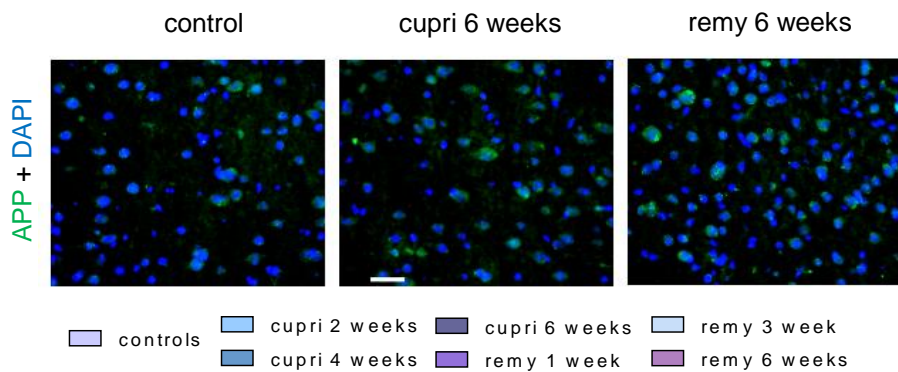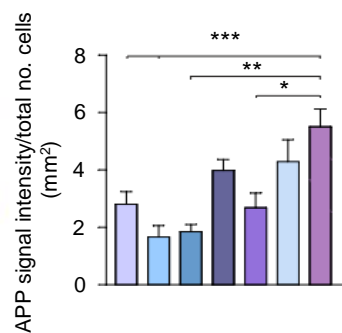

Supplement: Supplementary file 5 — Additional file 5: Figure S5. The amyloid precursor protein (APP) accumulation upon de- and remyelination in frontal neocortical regions. Exemplary pictures show APP staining in the lower layers of frontal neocortical regions in control conditions (left), at 6 weeks after starting the cuprizone diet (cupri 6 weeks – full demyelination, middle), and at full remyelination 6 weeks after reintroduction of normal food (right). Note that an increase of positive cells, indicating an accumulation of APP in the neuronal soma, occurred slowly at the onset of the cuprizone diet to reach a significant threshold at remyelinating phases. On the right, bar graphs show the number of APP positive cells (cells/mm2) in Cx. [file 12974_2020_1827_MOESM5_ESM.pdf]

**A**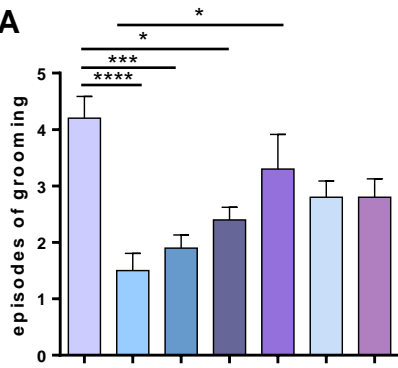

controls  
cupri 2 weeks  
cupri 4 weeks  
cupri 6 weeks  
remy 1 week  
remy 3 week  
remy 6 weeks

**B**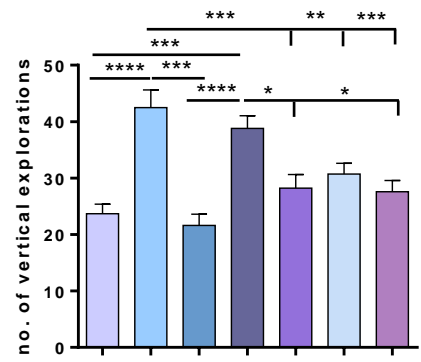

Supplement: Supplementary file 6 — Additional file 6: Figure S6. Exploratory and grooming behavior were altered by cuprizone diet and its withdrawn. (a) Bar graph showing quantification of vertical exploratory behavior. Animals show a significant increase in comparison to control 2- and 6 weeks after the beginning of the diet. (b) Bar graph showing the quantification of grooming behavior. The latter is often considered a indicator of stress levels I rodents and here it is significantly decreased, in comparison to control, in almost all experimental groups. *p < 0.05; **p < 0.01; ***p < 0.001; ****p < 0.0001. [file 12974_2020_1827_MOESM6_ESM.pdf]
